# Idiographic interrater reliability measures for intensive longitudinal multirater data

**DOI:** 10.1111/bmsp.70022

**Published:** 2025-12-20

**Authors:** Tobias Koch, Miriam F. Jaehne, Michaela Riediger, Antje Rauers, Jana Holtmann

**Affiliations:** ^1^ Friedrich‐Schiller‐Universität Jena Jena Germany; ^2^ Leipzig University Leipzig Germany

**Keywords:** empathic accuracy, idiographic rater consistency, longitudinal structural equation modeling, multirater analysis, time series analysis

## Abstract

Interrater reliability plays a crucial role in various areas of psychology. In this article, we propose a multilevel latent time series model for intensive longitudinal data with structurally different raters (e.g., self‐reports and partner reports). The new MR‐MLTS model enables researchers to estimate idiographic (person‐specific) rater consistency coefficients for contemporaneous or dynamic rater agreement. Additionally, the model allows rater consistency coefficients to be linked to external explanatory or outcome variables. It can be implemented in Mplus as well as in the newly developed R package mlts. We illustrate the model using data from an intensive longitudinal multirater study involving 100 heterosexual couples (200 individuals) assessed across 86 time points. Our findings show that relationship duration and partner cognitive resources positively predict rater consistency for the innovations. Results from a simulation study indicate that the number of time points is critical for accurately estimating idiographic rater consistency coefficients, whereas the number of participants is important for accurately recovering the random effect variances. We discuss advantages, limitations, and future extensions of the MR‐MLTS model.

## 1 BACKGROUND

The degree to which different raters converge in their assessment of a person's traits plays a critical role in various areas of psychological research (Funder, 1999; Hall et al., 2016; Kenny, 2019; Malloy, 2018). Numerous formulas exist for computing different types of interrater reliability (IRR). Among the most prominent measures of IRR are intraclass correlation coefficients (ICCs). These coefficients quantify the proportion of variance in the observed ratings that are shared by different raters judging a target person. It has been shown that the ICC aligns with the general definition of reliability in classical test theory (see ten Hove et al., 2022). Furthermore, different ICCs have been proposed for different research designs and research questions (see Gwet, 2014; Hallgren, 2012; Shrout & Fleiss, 1979, for an overview). More recently, the guidelines for selecting appropriate IRR measures have been updated to account for incomplete and dependent data (ten Hove et al., 2024a, 2024b).

Although ICC measures are well established for assessing interrater reliability in cross‐sectional designs, their adaptation to intensive longitudinal designs involving multiple raters (e.g., self‐reports and partner reports) remains less clear. However, many researchers aim to compute rater consistency coefficients in such measurement designs, as they enable a more fine‐grained analysis of rater consistency over time. Additionally, structural equation models (SEMs) offer a flexible way for computing rater consistency coefficients that are free from measurement error. SEMs also allow researchers to link latent variables as well as parameters associated with rater consistency coefficients to external variables. To date, only a few studies have examined how person‐specific reliability coefficients can be estimated within structural equation models (e.g., Hu et al., 2016; Schuurman & Hamaker, 2019; Xiao et al., 2023), but this work has not addressed the challenges posed by multirater designs.

To assess rater consistency in intensive longitudinal measurement designs, new models are needed that integrate the analysis of multirater data (i.e., multi‐method modeling) and multilevel latent time series modeling (e.g., dynamic structural equation modeling). These models allow for the computation of idiographic (i.e. person‐specific) rater consistency coefficients by modeling parameters associated with rater consistency as random parameters. In doing so, these models offer deeper insight into how accurately individuals can be judged by raters over time and in real‐world contexts.

A particularly relevant application of these models could be in the evaluation of empathic accuracy, which is defined as an individual's ability to correctly infer their partner's emotional state (Ickes, 1997; Riediger & Blanke, 2020). By its nature, empathic accuracy requires individuals to integrate and draw inferences from diverse sources of information available in everyday interactions (Blanke et al., 2015, 2016; Rauers et al., 2013). Since a partner's emotional state is not static but rather fluctuates over time, raters must continuously adjust their evaluations of the partner's emotional state. Measuring empathic accuracy over time requires a longitudinal dyadic or multirater design in which both the target and the partner complete self‐ and other‐report questionnaires regarding their affective states. In this context, idiographic rater consistency coefficients can be understood as a direct measure of a person's empathic accuracy. Building on this idea, our goal is to introduce rater consistency coefficients that capture psychological processes of empathic accuracy as they unfold over time. For simplicity, we will focus on the partner's ability to accurately judge the target's self‐reported affect. Note that the proposed model can easily be adapted to examine the target's ability to judge the partner's affect, or both. For such adaptations, we refer readers to the comprehensive textbook on structural equation modeling of multirater data by Eid et al. (2025).

The aim of this study is to propose new measures of person‐specific rater consistency for intensive longitudinal multirater designs with structurally different raters. Our framework adopts a design‐oriented modeling approach (Eid et al., 2016, 2025) and builds on dynamic structural equation modeling (DSEM; Asparouhov et al., 2018) as well as related multilevel latent time series models (e.g., Koslowski et al., 2024). In line with the design‐oriented modeling approach, we distinguish between fixed (or structurally different) and random (or interchangeable) raters, which leads to different models and rater consistency coefficients (see Eid et al., 2016, 2025; Koch et al., 2018, for details). This distinction parallels Kenny's (2019) differentiation between fixed and interchangeable raters as well as the distinctions commonly made when computing different ICCs for designs with fixed versus random raters (Shrout & Fleiss, 1979). In this study, we focus on intensive longitudinal measurement designs with fixed (or structurally different) raters, which represent the typical case in psychological research. However, the modeling approach proposed in this article can also be extended to measurement designs with interchangeable (random) raters (e.g., multiple students rating professors' teaching skills).

The article is organized as follows. First, we introduce the multirater latent state‐trait model with autoregressive effects (MR‐LST‐AR; Bohn et al., ( 2021 )) as a multilevel time series model with random parameters, including random loadings, random autoregressive effects, and random innovation variances. We explain how this new model enables the computation of idiographic interrater reliability coefficients at the within‐person level. Next, we illustrate the model using real data from an intensive longitudinal multirater study conducted in Germany, which involved 100 heterosexual couples (200 individuals) measured at 86 time points (Rauers et al., 2013). We also provide code for fitting the model in both Mplus (Muthén & Muthén, 1998–2017) and in R using the newly developed mlts package (Koslowski et al., 2024). Finally, we present a small simulation study as a sensitivity analysis to evaluate whether the parameter estimates are trustworthy in such applications.

## 2 MULTIRATER MULTILEVEL LATENT TIME SERIES MODEL

Bohn et al. (2021) introduced a multirater latent state‐trait model with autoregressive effects (MR‐LST‐AR) to analyze stability and change in dyadic attachment. The original MR‐LST‐AR was proposed as a single‐level structural equation model. The MR‐LST‐AR model integrates modern latent state‐trait theory (Eid et al., 2017; Steyer et al., 2015) with a design‐based multirater modeling approach (Eid et al., 2003, 2025; Koch et al., 2018), enabling researchers to quantify the degree of rater consistency between structurally different rater groups (here: target's self‐ratings and partner's ratings of the target) at three levels: the trait level, the momentary level, and the dynamic level. Recently, Koch et al. (2023) extended the MR‐LST‐AR model to a continuous‐time structural equation model. However, this extension focused solely on nomothetic rater consistency coefficients.

Here, we present the **MR**‐LST‐AR as a **M**ultilevel **L**atent **T**ime **S**eries (MR‐MLTS) model, which is suited for the analysis of intensive longitudinal data and allows for the computation of idiographic rater consistency coefficients. The basic idea of the model is to contrast a non‐reference rater group (here: the partner's ratings of the target person) against a reference rater group (here: the target's self‐ratings). The MR‐LST‐AR model is a DSEM model that is extended to include and relate different raters' perspectives on a construct of interest. Accordingly, main features described in the following correspond to models from the DSEM framework (Asparouhov et al., 2018), while the convergence between raters across time is modeled according to the approach used in Bohn et al. (2021).

### 2.1 Basic decomposition & measurement structure

In the following, we introduce the MR‐MLTS model step‐by‐step, referring to an individual rating as $y_{jk,it}$, where j denotes the indicator (e.g., an affect item), k refers to the rater group (target's self‐rating or partner rating), i refers to an individual (or dyad), and t to the time point. First, in line with standard multilevel time series models (see, e.g., Asparouhov et al., 2018), each rating associated with a rater group k is decomposed into a random intercept parameter $\mu_{jk,i}$ and a latent (person‐mean centered) within‐person variable $y_{jk,it}^{w}$. For three indicators per rater group (with k=1 denoting the target's self‐rating and k=2 denoting the partner rating), the following decomposition holds:

(1) $$\left[\begin{array}{c} y_{11,it}\\ y_{21,it}\\ y_{31,it}\\ y_{12,it}\\ y_{22,it}\\ y_{32,it} \end{array}\right]=\left[\begin{array}{c} \mu_{11,i}\\ \mu_{21,i}\\ \mu_{31,i}\\ \mu_{12,i}\\ \mu_{22,i}\\ \mu_{32,i} \end{array}\right]+\left[\begin{array}{c} y_{11,it}^{w}\\ y_{21,it}^{w}\\ y_{31,it}^{w}\\ y_{12,it}^{w}\\ y_{22,it}^{w}\\ y_{32,it}^{w} \end{array}\right]$$

The random intercept parameters $\mu_{jk,i}$ represent indicator‐specific stable trait (or latent person mean) variables, whereas the latent within‐person variables $y_{jk,it}^{w}$ capture momentary deviations of $y_{jk,it}$ from these latent person means at time point t. The decomposition of the observed variables according to Equation 1 is displayed in Figure 1a. Next, the latent within variables $y_{jk,it}^{w}$ are decomposed as follows at the within‐person level:

(2) $$\left[\begin{array}{c} y_{11,it}^{w}\\ y_{21,it}^{w}\\ y_{31,it}^{w}\\ y_{12,it}^{w}\\ y_{22,it}^{w}\\ y_{32,it}^{w} \end{array}\right]=\left[\begin{array}{cc} \lambda_{\text{O}11,i}^{w} & 0\\ \lambda_{\text{O}21,i}^{w} & 0\\ \lambda_{\text{O}31,i}^{w} & 0\\ \lambda_{\text{O}12,i}^{w} & \lambda_{\text{OR}12,i}^{w}\\ \lambda_{\text{O}22,i}^{w} & \lambda_{\text{OR}22,i}^{w}\\ \lambda_{\text{O}32,i}^{w} & \lambda_{\text{OR}32,i}^{w} \end{array}\right]\left[\begin{array}{c} O_{1,it}^{w}\\ OR_{2,it}^{w} \end{array}\right]+\left[\begin{array}{c} \varepsilon_{11,it}^{w}\\ \varepsilon_{21,it}^{w}\\ \varepsilon_{31,it}^{w}\\ \varepsilon_{12,it}^{w}\\ \varepsilon_{22,it}^{w}\\ \varepsilon_{32,it}^{w} \end{array}\right],$$

with $\varepsilon_{jk,it}^{w}∼N(0,\sigma_{\varepsilon_{jk}^{w}}^{2})$, for all i,t.

**FIGURE 1:**
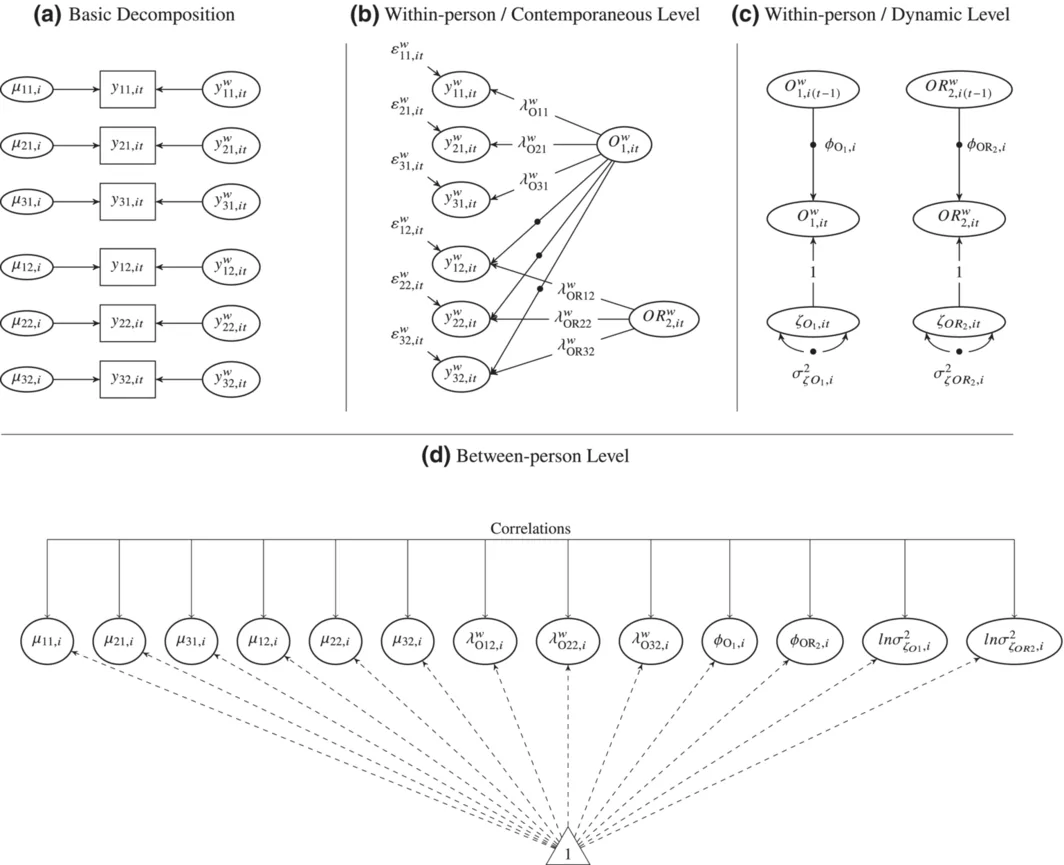
Path Diagram of the MR‐MLTS model. (a) Basic decomposition of observed variables. (b) Model at the momentary within‐person level. (c) Model at the dynamic within‐person level. (d) Model at the between‐person level. $y_{jk,it}$, observed variables; $\mu_{jk,i}$, random intercept variables; $y_{jk,it}^{w}$, latent within‐person variable; $O_{1,i(t-1)}^{w}$, occasion‐specific variable (reference rater group); $OR_{2,i(t-1)}^{w}$, occasion‐specific rater variable (non‐reference rater group); $\lambda_{\text{O}j1}^{w}$, fixed loadings of the occasion‐specific variable; $\lambda_{\text{OR}j2}^{w}$, fixed loadings of the occasion‐specific rater variable; $\lambda_{\text{O}j2,i}^{w}$, random cross‐loadings; $\phi_{{\text{O}}_{1},i}$, random AR effect pertaining to reference rater group; $\phi_{{\text{OR}}_{2},i}$, random AR effect pertaining to non‐reference rater group; $ln\sigma_{\zeta O_{1},i}^{2}$, random log innovation variance (reference rater group); $ln\sigma_{\zeta OR_{2},i}^{2}$, random log innovation variance (non‐reference rater group); j, indicator; k, rater‐group; i, person; t, time.

According to Equation 2, the latent within variables $y_{jk,it}^{w}$ pertaining to the partner rating (i.e., k≠1) are decomposed into a weighted occasion‐specific factor $\lambda_{\text{O}jk,i}^{w}O_{k,it}^{w}$, a weighted occasion‐specific rater factor $\lambda_{\text{OR}jk,i}^{w}OR_{k,it}^{w}$, and a measurement error variable $\varepsilon_{jk,it}^{w}$. In contrast, the decomposition of the $y_{jk,it}^{w}$‐variables pertaining to the target's self‐rating (i.e. k=1) does not include an occasion‐specific rater factor. This approach is known as the correlated trait‐correlated method minus one [CTC(M‐1)] approach in multimethod research (see Eid et al., 2016, 2025, 2003, 2008).

The CTC(M‐1) approach is similar to a latent linear regression approach, where the occasion‐specific factor of the target (here: reference rater group) serves as the independent variable to predict the occasion‐specific factor of the partner (here: non‐reference rater group). The residuals of this latent regression analysis are defined as occasion‐specific rater factors, denoted by $OR_{k,it}^{w}$. Specifically, the occasion‐specific rater factors $OR_{2,it}^{w}$ represent the unexplained true variance in the partner's ratings of the target's momentary affect that is not shared by the target's self‐rating. Consequently, the $O_{1,it}^{w}$‐variables and the $OR_{2,it}^{w}$‐variables are necessarily uncorrelated.

In Figure 1b, we illustrate the factor structure presented in Equation 2 for a specific person i at time t. As shown in Figure 1b and Equation 2, we allow the cross‐loadings of the occasion‐specific factor $O_{1,it}^{w}$ that pertain to the non‐reference rater group ($\lambda_{\text{O}12,i}^{w}$ to $\lambda_{\text{O}32,i}^{w}$; i.e., latent regression coefficients according to the CTC(M‐1) approach) to vary across dyads i. This approach is analogous to allowing for random (or person‐specific) latent regressions when predicting the occasion‐specific affect of the partner based on the occasion‐specific affect of the target. As a result, it becomes possible to calculate person‐specific rater consistency coefficients, since the amount of shared variance between target‐ and partner‐reported momentary affect ratings can vary across dyads. The variability in the cross‐loadings (or latent regression coefficients) is modeled at the between‐person level.

### 2.2 Measurement invariance

Measurement invariance is crucial for a meaningful interpretation of the parameters and rater consistency coefficients in the MR‐LST‐AR model. Four levels of measurement invariance are typically distinguished (Putnick & Bornstein, 2016) configural, weak, strong and strict. Below, we discuss the possible constraints on the factor loadings to establish weak measurement invariance across time, dyads (or individuals), and rater groups (e.g. self‐reports and partner reports). Firstly, to establish weak measurement invariance across time and meaningfully interpret the dynamic parameters in the model, all factor loadings must be set equal across time or at least certain time periods of the study. Secondly, to meaningfully compare idiographic rater consistencies across dyads, the loadings $\lambda_{\text{O}11,i}^{w}$ to $\lambda_{\text{O}31,i}^{w}$ for factor $O_{1,it}^{w}$ and $\lambda_{\text{OR}12,i}^{w}$ to $\lambda_{\text{OR}32,i}^{w}$ for factor $OR_{2,it}^{w}$ should be equal across dyads. If these loadings vary across dyads, it is still possible to compute rater consistencies. However, it is important to note that the coefficients are truly idiographic (i.e., dyadic‐specific), and only within‐dyads comparisons are warranted. By constraining these loading parameters to equality across dyads, we assume weak measurement invariance across dyads with respect to the factors $O_{1,it}^{w}$ and $OR_{2,it}^{w}$ (McNeish et al., 2021). Thereby, we make sure that the latent variables $O_{1,it}^{w}$ and $OR_{2,it}^{w}$ have the same substantive meaning across dyads, ensuring comparability of the rater consistency coefficients across dyads in the empirical applications. Note that the factor loadings which capture the latent regression of the partner ratings (non‐reference rating) on the target ratings (reference ratings) can be modeled as random effects (i.e., they are explicitly allowed to vary across dyads). Third, when multiple rater groups are available per dyad (e.g., self‐report, partner report, best‐friend report), weak measurement invariance should be established across rater groups (Eid et al., 2025). Establishing such invariance enables meaningful comparisons of rater consistency coefficients across rater groups. For example, one may examine whether the rater consistency between self and partner reports exceeds that between self and best‐friend reports. Weak measurement invariance across rater groups is established if the following parameter constraints hold $\lambda_{\text{O}j1}^{w}=\lambda_{\text{OR}j2}^{w}=\lambda_{\text{OR}jk}^{w}=\lambda_{\text{OR}j}^{w}$. When full measurement invariance cannot be established for all items, researchers may aim for partial measurement invariance (Byrne et al., 1989), requiring at least two invariant items per factor. However, achieving partial invariance is often difficult in intensive longitudinal designs with few (e.g., two) items per factor. Another challenge is testing measurement invariance using appropriate model‐fit statistics in multilevel latent time‐series models.

### 2.3 Dynamic level

At the dynamic within‐dyad level, the occasion‐specific (rater) variables are related over time via autoregressions:

(3) $$\left[\begin{array}{c} O_{1,it}^{w}\\ OR_{2,it}^{w} \end{array}\right]=\left[\begin{array}{cc} \phi_{{\text{O}}_{1},i} & 0\\ 0 & \phi_{{\text{OR}}_{2},i} \end{array}\right]\left[\begin{array}{c} O_{1,i(t-1)}^{w}\\ OR_{2,i(t-1)}^{w} \end{array}\right]+\left[\begin{array}{c} \zeta_{O_{1},it}\\ \zeta_{OR_{2},it} \end{array}\right],$$

with $\zeta_{O_{1},it}∼N(0,\sigma_{\zeta_{O_{1}},i}^{2})$ and $\zeta_{OR_{2},it}∼N(0,\sigma_{\zeta_{OR_{2}},i}^{2})$.

Equation 3 states that the occasion‐specific as well as the occasion‐specific rater variables are linked over time via autoregressive processes of order 1. These temporal relations are depicted in Figure 1c. Again, the autoregressive coefficients ($\phi_{{\text{O}}_{1},i}$ and $\phi_{{\text{OR}}_{2},i}$) are allowed to vary across dyads, as denoted by the index i. The autoregressive coefficient $\phi_{{\text{O}}_{1},i}$ captures the temporal stability (or carry‐over effects) of momentary affect as measured by the target's self‐rating. In contrast, the autoregressive coefficients $\phi_{{\text{OR}}_{2},i}$ are partial temporal stability coefficients, which capture carry‐over effects of momentary affect specific to the partners' perspectives after accounting for the targets' self‐ratings. In principle, it is also possible to specify cross‐lagged effects between the occasion‐specific variable representing the target's self‐report and the occasion‐specific rater variable capturing the partner's rater effect. However, the interpretation of these parameters is less straightforward and their inclusion may influence the estimation of other model parameters. If cross‐lagged effects are of substantive interest, we recommend the Actor–Partner Interdependence Model (APIM) as described by Savord et al. (2023).

The state residuals or innovations $\zeta_{O1,it}$ and $\zeta_{OR_{2},it}$ represent momentary affect fluctuations that cannot be predicted from previous measurements. The state residuals/innovations are assumed to follow a normal distribution with a mean of zero, respectively, with variances that can also vary across clusters (here: dyads) i. The state residuals of the reference ($\zeta_{O_{1},it}$) and non‐reference rater group ($\zeta_{OR_{2},it}$) are uncorrelated at each time point t (see definition of the variables in the CTC(M‐1) model in Eid et al., 2003, 2008). The person‐specific variance of the state residuals (innovation variance) and the autoregressive effects may correlate at the between‐person level. In DSEM, it is typical to model the natural logarithm of state‐residual (innovation) variances at the between‐person level. This transformation ensures positivity of the variances and simplifies the estimation process (Asparouhov et al., 2018).

### 2.4 Between‐person level

At the between‐person level, all random effects (i.e., random intercepts, loadings, autoregressive effects, and the logarithm of the state‐residual variances) are decomposed into an overall mean (fixed effect) and a person‐specific deviation:

(4) $$\left[\begin{array}{c} \mu_{11,i}\\ \mu_{21,i}\\ \mu_{31,i}\\ \mu_{12,i}\\ \mu_{22,i}\\ \mu_{32,i}\\ \lambda_{\text{O}12,i}^{w}\\ \lambda_{\text{O}22,i}^{w}\\ \lambda_{\text{O}32,i}^{w}\\ \phi_{{\text{O}}_{1},i}\\ \phi_{{\text{OR}}_{2},i}\\ \ln(\sigma_{\zeta O_{1},i}^{2})\\ \ln(\sigma_{\zeta OR_{2},i}^{2}) \end{array}\right]=\left[\begin{array}{c} \gamma_{0,\mu_{11}}\\ \gamma_{0,\mu_{21}}\\ \gamma_{0,\mu_{31}}\\ \gamma_{0,\mu_{12}}\\ \gamma_{0,\mu_{22}}\\ \gamma_{0,\mu_{32}}\\ \gamma_{0,\lambda_{\text{O}12}^{w}}\\ \gamma_{0,\lambda_{\text{O}22}^{w}}\\ \gamma_{0,\lambda_{\text{O}32}^{w}}\\ \gamma_{0,\phi_{\text{O}}}\\ \gamma_{0,\phi_{\text{OR}}}\\ \gamma_{0,\ln(\sigma_{\zeta O}^{2})}\\ \gamma_{0,\ln(\sigma_{\zeta OR}^{2})} \end{array}\right]+\left[\begin{array}{c} \upsilon_{\mu_{11,i}}\\ \upsilon_{\mu_{21,i}}\\ \upsilon_{\mu_{31,i}}\\ \upsilon_{\mu_{12,i}}\\ \upsilon_{\mu_{22,i}}\\ \upsilon_{\mu_{32,i}}\\ \upsilon_{\lambda_{\text{O}12,i}}\\ \upsilon_{\lambda_{\text{O}22,i}}\\ \upsilon_{\lambda_{\text{O}32,i}}\\ \upsilon_{\phi_{\text{O},i}}\\ \upsilon_{\phi_{\text{OR},i}}\\ \upsilon_{\ln(\sigma_{\zeta O}^{2}),i}\\ \upsilon_{\ln(\sigma_{\zeta OR}^{2}),i} \end{array}\right],$$

with $\upsilon_{i}∼MVN(0,\Omega )$ and Ω representing the unrestricted variance‐covariance matrix of the random effects. Figure 1d illustrates the between‐person model.

We recommend estimating all variances and covariances in Ω, as long as the resulting parameters are trustworthy (e.g., acceptable credibility intervals, reasonable posterior standard deviations, and no convergence issues of the Markov‐Chain Monte‐Carlo (MCMC) chains; see application section below). This corresponds to the least restrictive model that allows researchers to study heterogeneity among the indicators (i.e., indicator‐specificity) as well as rater‐consistency at the trait level. In the empirical illustration, we will provide a detailed discussion of the interpretation of covariances (or correlations) between random effects.

Researchers may also specify a factor structure for the random effects. For example, they may adopt a factor structure for the random intercepts that is similar to that used at the within‐person level, by regressing the random intercepts of the reference rater group (here: target's self‐ratings) on the random intercepts of the non‐reference rater group (here: partner rating of the target person). This approach allows for the specification of rater‐specific factors at the between‐person level. Similarly, researchers may consider modeling common factors for all random intercepts or random loadings. However, we do not recommend this approach, as affect measures typically consist of lists of adjectives (e.g., content, happy, relaxed), which typically lead to indicator‐specific effects that are not adequately captured when assuming a common factor model.

### 2.5 Idiographic rater consistency coefficients

The main advantage of the MR‐MLTS model is that it allows researchers to compute idiographic rater consistency coefficients. These can be defined for (a) contemporaneous consistency of ratings, (b) time‐lagged rater consistency, and (c) for rater consistency regarding the innovations. All three coefficients are conceptualized as between‐level coefficients, that is, they are dyad‐specific but constant across time. Below, we provide details on the definition and interpretations of the different coefficients. Rater‐consistency coefficients will be computed for each affect item, to facilitate a fine‐grained analysis and the selection of items with a high degree of rater consistency.

Rater consistency coefficients are conceptualized as variance coefficients, that is, they capture the degree to which differences in partner ratings are explained by differences in self‐ratings across time in percent of explained variance. Therefore, we first look at the model‐implied variance decomposition in the latent within‐level partner and self‐rating variables. According to Equations (1), (2), (3), the variance of the latent within variables $y_{jk,it}^{w}$ after accounting for measurement error, that is,

$$y_{jk,it}^{*w}=y_{jk,it}^{w}‐\varepsilon_{jk,it}^{w}$$

pertaining to the target's self‐rating (k=1) and the partner rating (k≠1) are given by:

(5) $$\begin{array}{ll} Var(y_{j1,i}^{*w}) & ={\left(\lambda_{\text{O}j1,i}^{w}\right)}^{2}\cdot \sigma_{O_{1},i}^{2} \end{array}$$

(6) $$\begin{array}{ll} Var(y_{jk,i}^{*w}) & ={\left(\lambda_{\text{O}jk,i}^{w}\right)}^{2}\cdot \sigma_{O_{1},i}^{2}+{\left(\lambda_{\text{OR}jk,i}^{w}\right)}^{2}\cdot \sigma_{OR_{k},i}^{2}\;\;\;,\;\;\;\;\;k\neq 1 \end{array}$$

at all time points (dropping the index *t* for variance(s) (components) in the following). Note that according to the AR(1) model, for the variances of $O_{1,i}$ and $OR_{k,i}$ it holds that $\sigma_{O_{1},i}^{2}=\frac{\sigma_{\zeta O_{1},i}^{2}}{1-\phi_{{\text{O}}_{1},i}^{2}}$ and $\sigma_{OR_{k},i}^{2}=\frac{\sigma_{\zeta OR_{k},i}^{2}}{1-\phi_{{\text{OR}}_{k},i}^{2}}$ (Hamilton, 1994). Therefore, the variances $\sigma_{O_{1},i}^{2}$ and $\sigma_{OR_{k},i}^{2}$ in Equations 5 and 6 can be further decomposed into a variance part that is carried over from the previous time point (time‐lagged, explained variance) and an innovation component:

(7) $$\begin{array}{ll} \sigma_{O_{1},i}^{2} & ={\left(\phi_{{\text{O}}_{1},i}\right)}^{2}\cdot \sigma_{O_{1},i}^{2}+\sigma_{\zeta O_{1},i}^{2}\; \end{array}$$

(8) $$\begin{array}{ll} \sigma_{OR_{k},i}^{2} & ={\left(\phi_{{\text{OR}}_{k},i}\right)}^{2}\cdot \sigma_{OR_{k},i}^{2}+\sigma_{\zeta OR_{k},i}^{2}\;\;,\;\;\;k\neq 1\; \end{array}$$

Contemporaneous rater consistency reflects the degree to which a partner rating can be explained by the target's self‐rating at the same time point, and thereby captures the overall contemporaneous convergence between the two ratings within time points:

(9) $$\begin{array}{ll} RCon(y_{jk,i}^{w}) & =\frac{{\left(\lambda_{\text{O}jk,i}^{w}\right)}^{2}\cdot \sigma_{O_{1},i}^{2}}{{\left(\lambda_{\text{O}jk,i}^{w}\right)}^{2}\cdot \sigma_{O_{1},i}^{2}+{\left(\lambda_{\text{OR}jk,i}^{w}\right)}^{2}\cdot \sigma_{OR_{k},i}^{2}}\; \end{array}$$

$RCon(y_{jk,i}^{w})$ can be interpreted similarly to an ICC coefficient, indicating the proportion of reliable within‐person variance in the partner‐reported affect of the target that overlaps with the target's self‐rated affect across time. That is, an $RCon(y_{jk,i}^{w})$ value of, e.g., .4 means that 40% of the within‐level true score variance in the partner reported affect is explained by true differences in the self‐reported affect over time, while 60% of the variance is specific to the partner's perspective. Based on Equation 9 we can see that rater consistency is larger with smaller variance of the occasion‐specific rater factor $OR_{k,it}^{w}$, that is, with smaller variance of the deviations of the other ratings from the self‐ratings.

For a more fine‐grained analysis, rater consistency can be calculated separately for the time‐lagged / predictable component of affect ratings and for the innovation components, analogous to the variance decomposition in Equations 7 and 8. For the time‐lagged (or autoregressive) component, rater consistency reflects the degree to which intraindividual dynamics in momentary affect in terms of carry‐over effects from previous time points, as measured by the partner rating, can be explained by the intraindividual carry‐over effects of the target's affect (for all k≠1):

(10) $$\begin{array}{ll} RConLag(y_{jk,i}^{w}) & =\frac{{\left(\lambda_{\text{O}jk,i}^{w}\right)}^{2}\cdot {\left(\phi_{{\text{O}}_{1},i}\right)}^{2}\cdot \sigma_{O_{1},i}^{2}}{{\left(\lambda_{\text{O}jk,i}^{w}\right)}^{2}\cdot {\left(\phi_{{\text{O}}_{1},i}\right)}^{2}\cdot \sigma_{O_{1},i}^{2}+{\left(\lambda_{\text{OR}jk,i}^{w}\right)}^{2}\cdot {\left(\phi_{{\text{OR}}_{k},i}\right)}^{2}\cdot \sigma_{OR_{k},i}^{2}}\; \end{array}$$

$RConLag(y_{jk,i}^{w})$ quantifies rater consistency with respect to the reliable variance that persists over time, i.e., is predictable by previous measurement time points. An $RConLag(y_{jk,i}^{w})$ value of, e.g., .4 means that 40% of the reliable variance in the partner‐reported affect that persists over time is explained by persistence in the self‐reported affect over time. Holding everything else constant, $RConLag(y_{jk,i}^{w})$ approaches one when the autoregressive effect of the rater factor $\phi_{OR_{k,i}}$ approaches zero. That is, when the rater's *misperception* of the target's self‐perception at the last measurement occasion is not carried forward in time, this is mirrored in a high $RConLag(y_{jk,i}^{w})$‐value. Analogously, a high $RConLag(y_{jk,i}^{w})$ coefficient suggests that the partner can detect intra‐individual persistence in the target's self‐reported affect over time.

For the innovation components, idiographic rater consistency indicates rater consistency with respect to the within‐dyad residual variances after accounting for time‐lagged effects:

(11) $$\begin{array}{ll} RConInn(y_{jk,i}^{w}) & =\frac{{\left(\lambda_{\text{O}jk,i}^{w}\right)}^{2}\cdot \sigma_{\zeta O_{1},i}^{2}}{{\left(\lambda_{\text{O}jk,i}^{w}\right)}^{2}\cdot \sigma_{\zeta O_{1},i}^{2}+{\left(\lambda_{\text{OR}jk,i}^{w}\right)}^{2}\cdot \sigma_{\zeta OR_{k},i}^{2}}\; \end{array}$$

$RConInn(y_{jk,i}^{w})$ represents the proportion of reliable within‐person variance in the partner‐reported innovations that is shared with the innovations of the self‐rating. It reflects the extent to which intra‐individual, unexplained fluctuations in the partner's ratings overlap with unexplained fluctuations in the self‐ratings. A high $RConInn(y_{jk,i}^{w})$ coefficient suggests high overlap between partner and self‐reported momentary affect deviations from the values that are predicted based on the previous time points' affect. Thereby, it reflects whether the partner can perceive unexpected changes in the target's affect from time point to time point. For instance, $RConInn(y_{jk,i}^{w})$ may be low when the partner has no access to information on the target's current affective state (e.g., within the last hour), such that the partner rating is based on the last available preceding information (e.g., from two hours ago), which is then only updated some time after his/her rating.

To investigate overall rater consistency, we can compute the average rater consistency coefficients across all dyads. Furthermore, the rater consistency coefficients can be linked to external variables to investigate which target or rater characteristics are associated with higher or lower rater agreement. Alternatively, primary model parameters such as random loadings ($\upsilon_{\lambda_{\text{O}jk,i}}$), random autoregressive effects ($\upsilon_{\phi_{\text{O},i}}$ and $\upsilon_{\phi_{\text{OR},i}}$), and random innovation variances ($\upsilon_{\ln(\sigma_{\zeta O}^{2}),i}$ and $\upsilon_{\ln(\sigma_{\zeta OR}^{2}),i}$) can be directly linked to external variables within the model, to explain heterogeneity in parameters that are associated with the idiographic rater consistency coefficients. Note, however, that between‐level correlations of, for instance, the random loading parameters with external variables use unstandardized loadings, which do not directly capture rater consistency as dyads may vary with respect to their variability in affect ratings across time.

### 2.6 Assumptions

The MR‐MLTS model is a time series model and assumes stationarity with regard to the latent occasion‐specific variables. This means that the following restrictions are imposed:

1. Equal loadings of the occasion‐specific factors $\lambda_{\text{O}j1t}^{w}=\lambda_{\text{O}j1t^{\prime }}^{w}=\lambda_{\text{O}j1}^{w}$ (for k=1) and occasion‐specific method factors $\lambda_{\text{OR}jkt}^{w}=\lambda_{\text{OR}jkt^{\prime }}^{w}=\lambda_{\text{OR}jk}^{w}$ (for k≠1) across time.
2. Equal variance of the state‐residual factors $\sigma_{\zeta O_{1},it}^{2}=\sigma_{\zeta O_{1},it^{\prime }}^{2}=\sigma_{\zeta O_{1},i}^{2}$ and state‐residual method factors $\sigma_{\zeta OR_{kt}}^{2}=\sigma_{\zeta OR_{kt^{\prime }}}^{2}=\sigma_{\zeta OR_{k}}^{2}$ across time. The variance of these factors may differ across persons.
3. Equal autoregressive effects $\phi_{\text{O}1,it}=\phi_{\text{O}1,it^{\prime }}=\phi_{\text{O}1,i}$ and $\phi_{\text{OR}k,it}=\phi_{\text{OR}k,it^{\prime }}=\phi_{\text{OR}k,i}$ across time. These autoregressive effects may differ across persons.
4. Equal measurement error variances $\sigma_{\varepsilon_{jkt}}^{2}=\sigma_{\varepsilon_{jkt^{\prime }}}^{2}=\sigma_{\varepsilon_{jk}}^{2}$ across time (and persons).

Assumption 1 states that the loadings of the occasion‐specific (method) factors are invariant across time, thereby assuming measurement invariance across time. Furthermore, these loadings are assumed to be invariant across persons (an assumption that may be relaxed, see section on measurement invariance).The cross‐loadings of the items pertaining to the non‐reference rater group on the occasion‐specific factors of the reference rater group, $\lambda_{\text{O}jk,i}^{w}$ with k≠1, are modeled as dyad‐specific, i.e., random across dyads. Assumption 2 states that the variances of the occasion‐specific (method) residuals are equal over time. Assumption 3 implies that the autoregressive effects are invariant over time, meaning there are no time‐varying autoregressive effects. Finally, Assumption 4 asserts that the measurement error variance remains constant across time. Furthermore, the models assume that there is no trend (i.e., no change in the mean level) across time in the data. It is worth noting that these assumptions are commonly made in time series models and are automatically incorporated when fitting the model in Mplus or mltssoftware.

## 3 APPLICATION OF THE MR‐MLTS MODEL

We illustrate the MR‐MLTS model using data from an intensive longitudinal multirater study conducted in Germany, involving 100 heterosexual couples (200 individuals in total) measured at 86 time points (Rauers et al., 2013). The sample consists of two age groups: 100 younger adults (ages 20–30, M = 25.94, SD = 2.94) and 100 older adults (ages 69–80, M = 74.20, SD = 2.89). The data contain both self‐ as well as other ratings for both the anchor persons and their partners. We focus on the assessment of positive affect as the dependent variable and present the results when using the anchor persons' self‐ratings as the reference rater group (target) and the partner ratings of the anchors' affect as the non‐reference rater group.^1^ We use three positive‐affect items (i.e., happy, enthusiastic, and content) on a 7‐point rating scale. Additionally, we linked the idiographic rater‐consistency coefficients to the time‐stable external variables relationship duration, target's emotional resources, and partner's cognitive resources, for illustrative purposes. Emotional competencies were measured using the targets' self‐rated attention to other people's feelings (Lischetzke et al., 2001) and cognitive‐mechanic resources of the partner were measured using the digit‐symbol substitution test (Wechsler, 1994).

We provide detailed code and examples to fit the MR‐MLTS model and compute consistency coefficients in both Mplus (Muthén & Muthén, 1998–2017) and R (R Core Team, 2024), the latter using the R‐packages mlts by Koslowski et al. (2024) and rstan (Stan Development Team, 2025a, 2025b). All models are estimated using Bayesian MCMC sampling, with the posterior medians serving as the parameters' point estimates. In the following, we present the results obtained using Mplus. To account for unequal time intervals between adjacent measurements we set the tinterval option in Mplus to two hours, as indicated as a reasonable value by the distribution of the observed time intervals in the data. To ensure convergence of the MCMC chains, we ran a minimum of 20,000 iterations with a thinning factor of 10 (resulting in a minimum of 200,000 iterations), targeting a stringent convergence criterion of a Potential scale reduction (PSR) factor below 1.02. We did not encounter any convergence issues, and sampling stopped at 20,000 iterations with a PSR of 1.002. Traceplots and autocorrelation plots can be found in the online supplemental materials at OSF. For model comparison, we provide the DIC and the estimated number of parameters (pD) from Mplus. The DIC was 139580.964 and the pD was 10225.591. Note that there are no established Bayesian model fit statistics for these types of models in Mplus.

To compute the consistency coefficients and their associations with external covariates, we saved 1000 plausible values from the parameters' posterior distributions.^2^ Consistency coefficients and their associations with between‐dyad covariates were then computed for each of these 1000 draws from the posterior distribution. The mean of the dyad‐specific consistency coefficients, serving as an estimate of the fixed effect γ, and the variance of the consistency coefficients across dyads, serving as an estimate of the random effect variance, were calculated per posterior draw and summarized across the 1000 draws. We report the median across the posterior draws as point estimates and the 2.5% and 97.5% quantiles as borders of 95% credibility intervals (CI).^3^

The same procedure is adopted for the regression models relating consistency coefficients to the targets' and raters' characteristics. All continuous explanatory variables (i.e., relationship duration, target's emotional resources, and partners' cognitive resources) were standardized and included as independent variables in the regression models predicting rater consistency. Note that in this application, we only use between‐level trait variables as covariates. To investigate the effect of time‐varying covariates on the autoregressive (AR) effects, innovation variances, or loading parameters (and thereby on the rater consistencies), an extension of the presented model to incorporate within‐level interaction effects is needed. This kind of model can be implemented and estimated using the mlts‐package (Koslowski et al., 2024). However, this is beyond the scope of the present article. See Holtmann and Koslowski (2025) for details on within‐level latent interaction models and their application.

For completeness, we also re‐estimated the model with the external covariates entered as between‐level covariates for the primary model parameters (i.e., random loadings, autoregressive effects, log innovation variances) in the Mplus model. The results of the latter model are available on OSF. All code is available on OSF at https://osf.io/g8hnz/?view_only=0df80fab09f74b249d4eeb7a56af1636.

## 4 RESULTS

In Table 1, we present unstandardized parameter estimates as well as average within‐person standardized parameter estimates from the model with anchor persons as reference raters (targets). For information on standardization of random within‐level parameters in DSEM‐type models see Schuurman, Ferrer, et al. (2016).

**TABLE 1:** Parameter estimates for the model with anchor persons as reference raters.

Unstandardized parameter estimates					
Parameter	Estimate	95% CI	Parameter	Estimate	95% CI
**Within‐person level (fixed parameters)**					
$\lambda_{\text{O}11}^{w}$	1.000	–	$\sigma_{\epsilon_{11}^{w}}^{2}$	.588	[.560; .618]
$\lambda_{\text{O}21}^{w}$	.850	[.820; .882]	$\sigma_{\epsilon_{21}^{w}}^{2}$	1.056	[1.018; 1.094]
$\lambda_{\text{O}31}^{w}$	.961	[.931; .991]	$\sigma_{\epsilon_{31}^{w}}^{2}$	.676	[.646; .706]
$\lambda_{\text{OR}12}^{w}$	1.000	–	$\sigma_{\epsilon_{12}^{w}}^{2}$	.512	[.484; .541]
$\lambda_{\text{OR}22}^{w}$	.873	[.820; .927]	$\sigma_{\epsilon_{22}^{w}}^{2}$	.774	[.742; .806]
$\lambda_{\text{OR}32}^{w}$	.859	[.812; .907]	$\sigma_{\epsilon_{32}^{w}}^{2}$	.674	[.647; .702]
**Between‐person level**					
*Fixed effects*			*Random effect variances*		
$\gamma_{\lambda_{\text{O}12}^{w}}$	.556	[.487; .630]	$\sigma_{\lambda_{\text{O}12}^{w}}^{2}$	.087	[.056; .138]
$\gamma_{\lambda_{\text{O}22}^{w}}$	.476	[.401; .554]	$\sigma_{\lambda_{\text{O}22}^{w}}^{2}$	.106	[.068; .167]
$\gamma_{\lambda_{\text{O}32}^{w}}$	.502	[.428; .500]	$\sigma_{\lambda_{\text{O}32}^{w}}^{2}$	.107	[.068; .168]
$\gamma_{\phi_{\text{O}}}$	.603	[.553; .652]	$\sigma_{\phi_{\text{O}}}^{2}$	.047	[.031; .071]
$\gamma_{\phi_{\text{OR}}}$	.599	[.531; .661]	$\sigma_{\phi_{\text{OR}}}^{2}$	.068	[.045; .103]
$\gamma_{\ln(\sigma_{\zeta \text{O}}^{2})}$	−.897	[‐1.182; −.622]	$\sigma_{\ln(\sigma_{\zeta \text{O}}^{2})}^{2}$	1.785	[1.228; 2.673]
$\gamma_{\ln(\sigma_{\zeta \text{OR}}^{2})}$	−1.771	[−2.148; −1.405]	$\sigma_{\ln(\sigma_{\zeta \text{OR}}^{2})}^{2}$	2.924	[2.028; 4.363]
**Within‐person standardized parameter estimates averaged across persons**					
$\lambda_{\text{O}12}^{w}$	.418	[.395; .443]	$\sigma_{\epsilon_{11}^{w}}^{2}$	.436	[.417; .455]
$\lambda_{\text{O}22}^{w}$	.337	[.313; .363]	$\sigma_{\epsilon_{21}^{w}}^{2}$	.630	[.611; .651]
$\lambda_{\text{O}32}^{w}$	.374	[.351; .399]	$\sigma_{\epsilon_{31}^{w}}^{2}$	.483	[.464; .503]
$\phi_{\text{O}}$	.603	[.577; .627]	$\sigma_{\epsilon_{12}^{w}}^{2}$	.459	[.436; .483]
$\phi_{\text{OR}}$	.600	[.559; .631]	$\sigma_{\epsilon_{22}^{w}}^{2}$	.603	[.580; .625]
$\sigma_{\zeta \text{O}}^{2}$	.600	[.573; .627]	$\sigma_{\epsilon_{32}^{w}}^{2}$	.568	[.548; .588]
$\sigma_{\zeta \text{OR}}^{2}$	.592	[.556; .633]			

*Note*: Estimates correspond to the posterior median of the respective parameter's posterior distribution.

Abbreviations: CI, credibility interval; ln, natural logarithm; O, occasion‐specific factor; OR, occasion‐specific rater factor; γ, fixed effect (average across clusters); ε, measurement error; ζ, innovation; $\lambda^{w}$, loadings; $\sigma^{2}$, variance; ϕ, autoregressive effects.

Table 2 provides fixed and random effect estimates for the idiographic rater consistency coefficients, descriptive statistics of their distribution across persons, as well as correlations between the different coefficients. The average rater consistency coefficients range from .372 to .413 across items, indicating a substantial overlap between self‐reported and partner‐reported affect ratings. These results suggest that, on average (across individuals), 37% to 41% of the true variance in partner‐reported affect ratings is shared with the self ratings, depending on the item. However, the rater consistency coefficients also exhibit considerable variability between individuals, with standard deviations ranging from .210 to .286. As shown in Table 2, the individual coefficients span from .007 (indicating no overlap) to .890 (indicating strong overlap) across persons. Figure 2 further illustrates the distribution of the idiographic time‐lagged and innovation rater consistency coefficients using violin plots. Overall, the results suggest that individuals differ strongly in their empathic accuracy.

**FIGURE 2:**
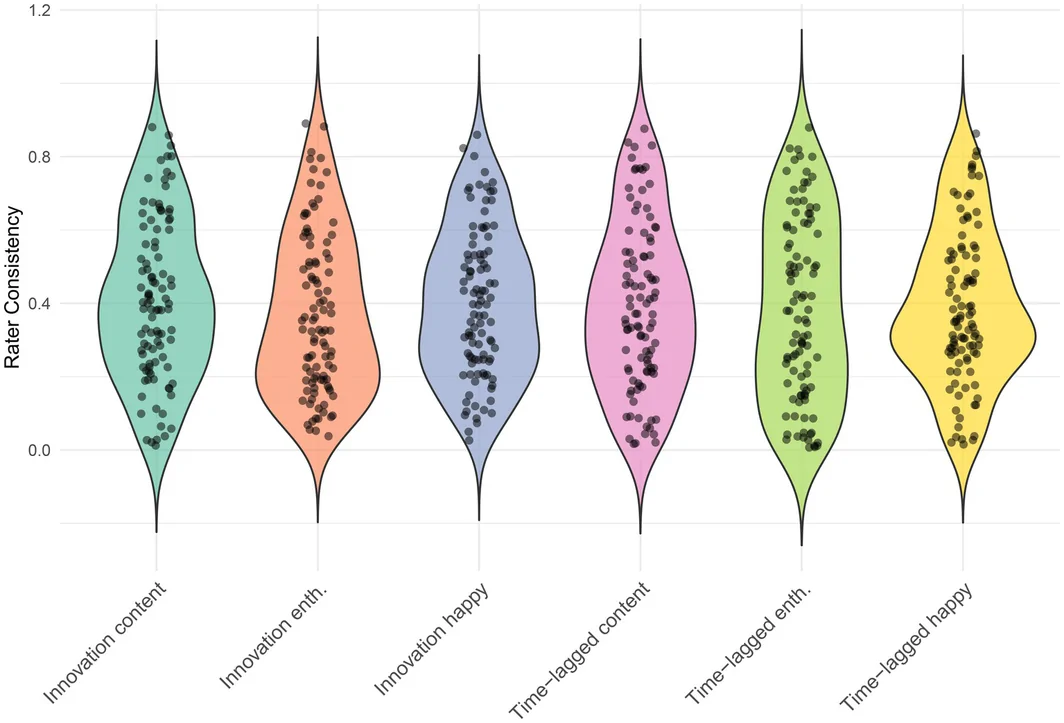
Violin Plot of Idiographic Rater Consistency Coefficients. Violin plots depict the distribution of individual rater consistency coefficients across individuals, for coefficients for the innovations as well as time‐lagged rater consistency, per item. enth., item enthusiastic.

**TABLE 2:** Descriptive statistics and correlations of idiographic rater consistency coefficients.

Item	Fixed effect		Random effect		Distribution across dyads			
	γ	95% CI	$\sigma^{2}$	95% CI	Min	2.5%	97.5%	Max
Contemporaneous rater consistency $\left(RCon(y_{jk,i}^{w})\right)$								
Happy	.372	[.333; .404]	.044	[.032; .071]	.061	.077	.699	.792
Enthusiastic	.357	[.321; .393]	.058	[.044; .079]	.028	.049	.749	.818
Content	.384	[.345; .422]	.056	[.043; .084]	.013	.046	.729	.759
Rater consistency for the innovations $\left(RConInn(y_{jk,i}^{w})\right)$								
Happy	.402	[.360; .444]	.059	[.038; .073]	.026	.079	.780	.860
Enthusiastic	.378	[.334; .419]	.066	[.045; .080]	.038	.062	.805	.890
Content	.413	[.366; .453]	.069	[.050; .081]	.012	.027	.817	.881
Time‐lagged rater consistency $\left(RConLag(y_{jk,i}^{w})\right)$								
Happy	.399	[.345; .451]	.065	[.047; .089]	.015	.028	.791	.863
Enthusiastic	.394	[.341; .442]	.082	[.063; .108]	.007	.011	.811	.879
Content	.404	[.350; .458]	.073	[.054; .098]	.017	.025	.829	.876

Correlations between rater consistency coefficients						
	1	2	3	4	5	6
1. Innov. hap.	1.00	[.576; .861]	[.671; .909]	[−.190; .336]	[−.294; .220]	[−.204; .312]
2. Innov. enth.	**.753**	1.00	[.406; .759]	[−.096; .401]	[.120; .534]	[−.106; 351]
3. Innov. cont.	**.812**	**.616**	1.00	[−.228; .260]	[−.299; .155]	[.028; .470]
4. Lagged hap.	.097	.163	.023	1.00	[.759; .930]	[.730; .926]
5. Lagged enth.	−.001	**.344**	−.059	**.866**	1.00	[.571; .841]
6. Lagged cont.	.067	.141	**.261**	**.852**	**.733**	1.00

*Note*: Values below the diagonal of the lower table represent the posterior median and values above the diagonal the 95% credibility interval for the Pearson correlation coefficient between the rater consistency coefficients. Correlation parameters with 95% CIs niot covering zero are printed in bold face.

Abbreviations: 95% CI, credibility interval with limits corresponding to the 2.5% and 97.5% posterior quantiles; cont., content; enth., enthusiastic; hap., happy; Innov., rater consistency for the innovations; Lagged, time‐lagged rater consistency; Max, maximum value across dyads; Min, minimum value across dyads; γ, posterior median of the average rater consistency coefficient across dyads; $\sigma^{2}$, posterior median of the variance of the rater consistency coefficients across dyads.

Table 2 also presents between‐dyad correlations and their CIs of the rater consistency coefficients for the time‐lagged and innovation components. The complete correlation matrix including the contemporaneous rater consistency coefficients is provided in supplementary material on OSF. Consistency coefficients are positively correlated between different items when referring to the same type of consistency. For example, the correlations between the time‐lagged rater consistency coefficients of different items range from .733 to .866, indicating that individuals' empathic accuracy generalizes across different affect measures (here: items). The correlations between the innovation rater consistency coefficients of different items are somewhat smaller, ranging from .616 to .812. This also holds true for the contemporaneous rater consistency coefficients, with correlations (not shown in Table 2) ranging between .603 and .787.

The time‐lagged and innovation rater consistency coefficients are largely unrelated, except for within‐item correlations, for instance for the item ‘enthusiastic’ (see Table 2). Correlations of the contemporaneous rater consistency with the other consistency coefficients are larger, ranging from .650 to .773 and from .337 to .570 for within‐item as well as between‐item correlations, respectively (see Tables in supplementary file on OSF). The larger correlations of contemporaneous rater consistency with the other rater consistency coefficients can be explained by their mathematical overlap; that is, time‐lagged as well as innovation components are both contained within contemporaneous rater consistency. In contrast, time‐lagged rater consistency and rater consistency for the innovations refer to independent variance components in the ratings.

Next, we related external explanatory trait variables to the rater consistency coefficients. As shown in Table 3, the explanatory variables did, with two exceptions, not have significant effects on the time‐lagged rater consistency coefficients. This may be partly explained by the fact that the predictor variables represent between‐level trait variables and do not capture time‐dependent, variable factors that might affect the persistence in ratings across time.

**TABLE 3:** Regression results for rater consistency coefficients of the innovations.

Predictor	Happy		Enthusiastic		Content	
	Est.	95% CI	Est.	95% CI	Est.	95% CI
Rater consistency coefficients for the innovations						
Intercept	**.402**	[.360; .444]	**.378**	[.334; .419]	**.413**	[.366; .453]
β: Relationship duration	**.071**	[.034; .120]	**.046**	[.006; .092]	.040	[−.002; .089]
β: Target's emot. resources	−.000	[−.026; .026]	−.000	[−.029; .028]	.018	[−.012; .048]
β: Partner's cogn. resources	**.066**	[.030; .106]	.027	[−.011; .072]	**.065**	[.024; .111]
$R^{2}$	.076	[.025; .165]	.030	[.004; .089]	.058	[.015; .135]
Time‐lagged rater consistency coefficients						
Intercept	**.399**	[.345; .451]	**.394**	[.341; .442]	**.404**	[.350; .458]
β: Relationship duration	−.028	[−.074; .017]	−.036	[−.083; .009]	**−.047**	[−.097; −.002]
β: Target's emot. resources	−.021	[−.058; .011]	−.023	[−.060; .015]	−.007	[−.044; .030]
β: Partner's cogn. resources	−.027	[−.070; .024]	**−.051**	[−.098; −.002]	−.027	[−.072; .025]
$R^{2}$	.029	[.003; .085]	.038	[.006; .094]	.030	[.004; .095]

*Note*: Parameters with 95% CIs not covering zero are printed in bold face.

Abbreviations: β, regression coefficients; 95% CI, Credibility interval with limits equal to 2.5% and 97.5% quantiles of posterior draws; cogn., cognitive; emot., emotional; Est., median estimate across 1000 draws from the parameter's posterior distribution.

For rater consistency of the innovation components, that is, for $RConInn(y_{jk,i}^{w})$, relationship duration was positively associated with higher rater consistency in the assessment of happiness and enthusiasm. This suggests that partners in longer relationships were better able to judge unexpected fluctuations in the targets’ happiness and enthusiasm as compared to partners from couples in shorter relationships. Additionally, the partner's cognitive resources were positively associated with greater innovation rater consistency for happiness and contentedness, reflecting a higher empathic accuracy of the partner (emphasizer) with respect to the target's affect in a specific moment. Note that in our analysis, rater consistency reflects the ability of the partner rather than that of the target person, due to the choice of the reference rater group.

In Figure 3, we display the correlations between the random effects at the between‐person level. Correlations between random intercepts (μ) within the same rater group (T for target's self‐ratings or P for partner ratings) but across different items reflect trait‐level heterogeneity between affect items (i.e., happy, enthusiastic, content). Higher correlations indicate lower heterogeneity (lower indicator specificity). In our study, these correlations ranged from .551 to .785 for the targets' self‐reports and from .560 to .797 for partner reports. This suggests a considerable amount of heterogeneity between the persons' average affect captured by the different items. These correlations can also be interpreted as discriminant validity coefficients, indicating the extent to which raters can distinguish between different affective states (e.g., happy vs. content).

**FIGURE 3:**
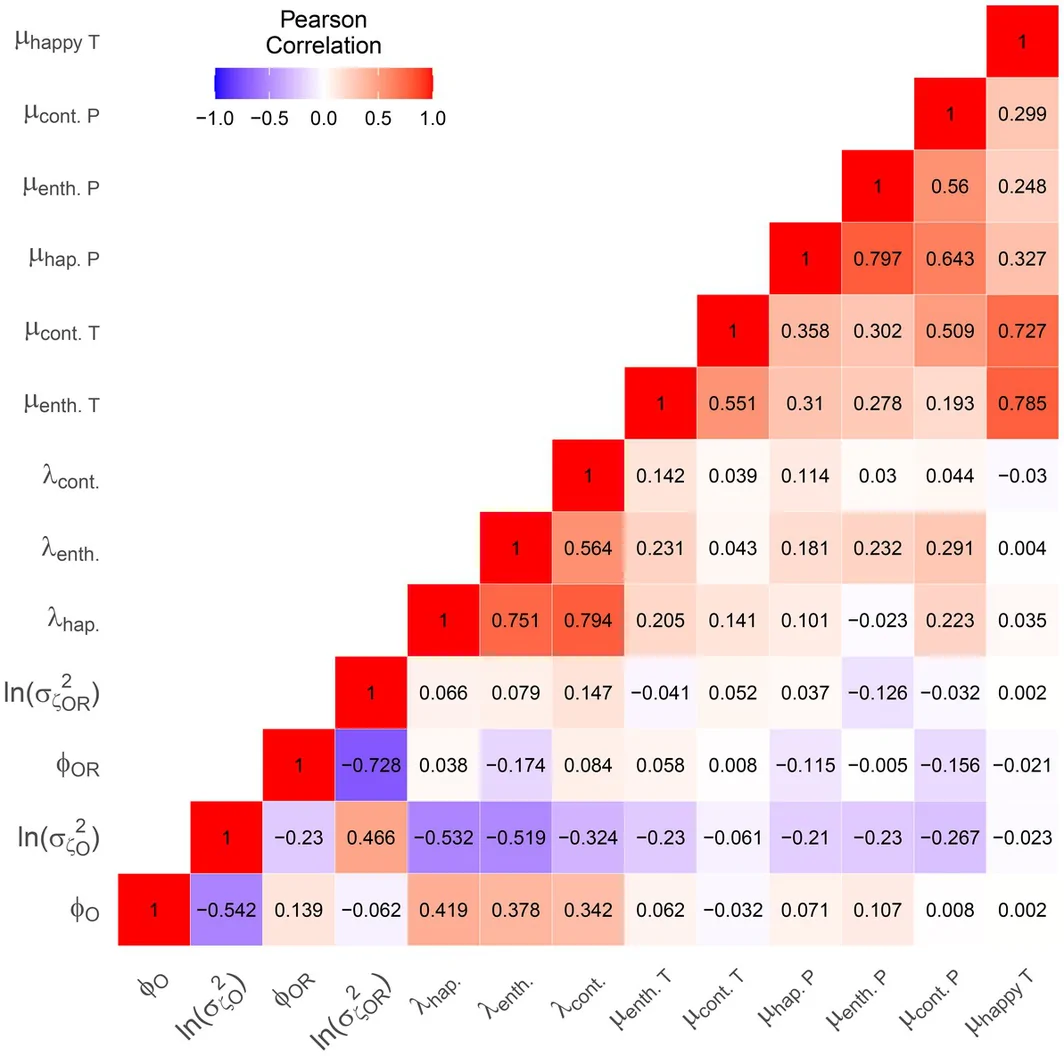
Heat Map of Between‐Level Correlations Between Random Effects. cont., item content; enth., item enthusiastic; hap., item happy; O, occasion‐specific factor; OR, occasion‐specific rater factor; P, partner rating; T, target's self‐rating; λ, random cross‐loading/latent regression coefficient from regressing non‐reference rating (partner rating) on the reference rating (target's self‐rating); μ, random intercept; $\ln(\sigma_{\zeta }^{2})$, random log‐innovation variance; ϕ, random autoregressive effect.

Correlations between the random intercepts (μ) belonging to the same observed variable but different rater groups can be interpreted as a measure of rater consistency at the trait level. Higher correlations suggest stronger overlap between the target's mean self‐ratings and mean partner ratings. The correlations vary between .327 (happy), .278 (enthusiastic), and .509 (content).

Correlations between the random loadings and the random intercepts have an interpretation analogous to those in mixed‐effects models. A positive correlation would indicate that individuals with a high level of positive affect (happiness) tend to show a stronger relationship between self‐ and other‐ratings (higher rater consistency). In our study, the correlations are generally low and close to zero, with few exceptions (see Figure 3).

Correlations between the random loadings and the random autoregressive effect of the self‐rating reveal the extent to which temporal stability in the targets' affect is linked to rater consistency. Interestingly, the random loadings were positively associated with the random autoregressive effects of the target's self‐ratings, suggesting that partners are better at judging targets who exhibit higher stability in their affect over time. Similarly, correlations between the random loadings and random innovation variances in the self‐ratings indicate the extent to which temporal instability (fluctuation) in the targets' affect over time is associated with rater consistency. The negative correlations suggest that targets with higher affective instability tend to be rated with lower rater consistency.

Correlations between random autoregressive effects (RAuto) and random innovation variance (RInno) within each rater group (target's self‐ratings and partner ratings) reflect the relationship between temporal stability and occasion‐specific fluctuations in momentary affect. Typically, these correlations are negative, indicating that higher temporal stability is associated with lower occasion‐specific fluctuations. In our study, these correlations were −.54 for the target's self‐rating and −.73 for the partner rating, indicating a strong negative relationship between temporal stability and occasion‐specific fluctuations in momentary affect for both rater groups.

Correlations between the autoregressive effects and random innovation variance across rater groups provide indicators for how dynamics in the targets' affect across time may influence the raters' perception of their affect and fluctuations therein. Overall, these correlations were relatively low and close to zero, with the exception of the correlation of the innovation variances across rater groups. The latter suggests that larger fluctuations in the target's self‐ratings are associated with larger fluctuations in the partner‐specific perspective (after accounting for the self‐rating). That is, targets with less variation in affect across time have raters with less variation in their rater‐specific effects across time, suggesting that the affective state of targets with less variable affect might be easier to perceive for others.

Correlations between the random autoregressive effects and innovation variances within rater groups show that, (a) targets with higher inertia in affect tend to also have lower innovation variances, and (b) raters with higher inertia in *misperceived* ratings (i.e., in their deviations from the self‐ratings) tend to have lower variability with respect to the innovations. The latter suggests that raters which rely more strongly on their last judgment tend to show less or smaller spontaneous variability in their rater effects.

With respect to the between‐level covariates, only relationship duration was associated with the innovation variance in both the self‐ratings of affect as well as the innovation variance in the rater‐specific perspective, suggesting less variability in affect for couples in longer relationships. Between‐level correlation coefficients along with their CIs for the model with between‐level covariates are presented in Table S1 in the supplemental material provided on OSF.

## 5 SIMULATION STUDY

We examined the estimation accuracy of the individual, person‐specific parameters and consistency coefficients in the MR‐MLTS model in a small proof of principle simulation study. The simulation study was designed as a sensitivity analysis to assess whether the rater consistencies can be reliably estimated in the setting of the present empirical application. We varied the number of dyads (N = 100, 200) and time points per dyad (T = 100, 200) with the aim to determine sufficiency of these sample sizes for accurate parameter estimation. For each condition, we simulated 100 datasets / replications, using values similar to the parameter estimates obtained in the application section as data generating values. All datasets were generated in R (R Core Team, 2024) and models were estimated using Mplus. Estimation accuracy was judged using relative parameter estimation bias, coverage rates, and the mean squared error (MSE). The primary parameter of interest in the present case is the reliability of the individual‐specific parameter estimates and consistency coefficients. Reliability of individual‐specific parameters / coefficients was calculated as the average correlation between the true data‐generating values and the estimated person‐specific parameters. All code files for replicating the data generation as well as the post‐processing of the results are provided on OSF.

In general, fixed effects γ (see Equation 4) are estimated accurately in all conditions (see Table S.2 in the supplemental material on OSF for detailed results). The same holds true for parameters of the measurement model. However, between‐level random effect variances of the $\nu_{i}$ are systematically overestimated across conditions, with decreasing bias with larger person sample sizes at the between‐person level.

Reliabilities for the individual‐specific person parameter estimates are depicted in Figure 4. Mean reliabilities are very good for the primary model parameters, with average reliabilities across replications for the autoregressive effects, innovation variances, and random loadings assuming values ≥.87 for T=100 and ≥.93 for T=200. Reliabilities are satisfactory for person‐specific consistency coefficients, with increasing reliability for a larger number of observed time points per person (average reliability ≥.80 for T=100 and ≥.88 for T = 200 time points).

**FIGURE 4:**
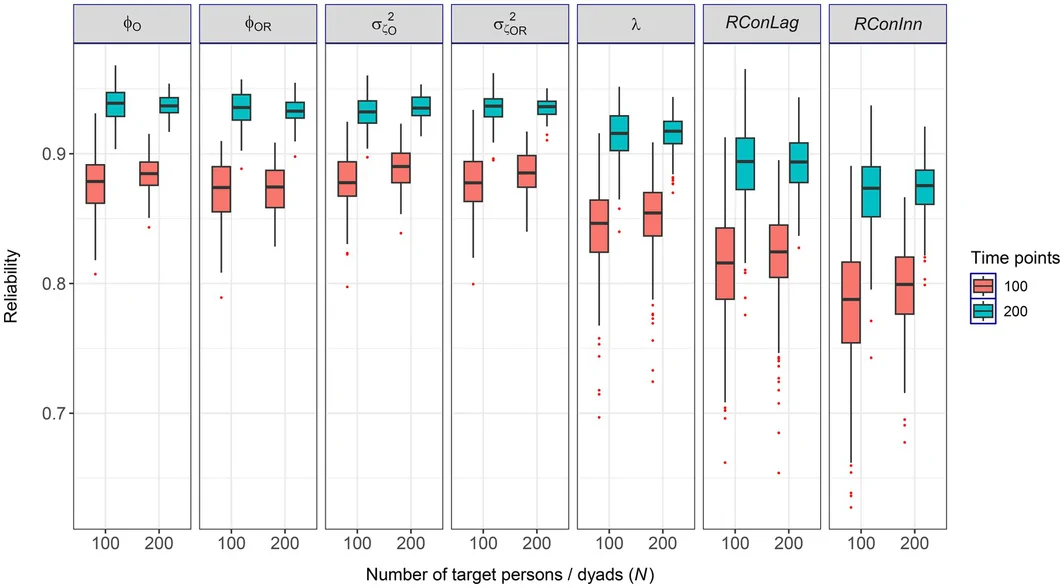
Reliabilities of person‐specific parameter estimates and rater consistency coefficients. Displayed are boxplots of the distribution of the individual‐specific parameters' reliability estimates across replications, as estimated with Mplus. Reliability estimates are displayed jointly for the same parameter type of different items. O, occasion‐specific factor; OR, occasion‐specific rater factor; RConInn, rater consistency coefficient for the innovation component; RConLag, time‐lagged rater consistency coefficient; $\sigma_{\zeta }^{2}$, logarithm of the innovation variance; λ, random cross‐loading/latent regression coefficient from regressing non‐reference rating (partner rating) on the reference rating (target's self‐rating); ϕ, autoregressive effect.

## 6 DISCUSSION

In this article, we propose a multilevel latent time series model for longitudinal multirater data, termed the MR‐MLTS model, to compute person‐specific rater consistency coefficients. This model extends the multirater latent state‐trait approach introduced by Bohn et al. (2021), facilitating the evaluation of rater consistency in intensive longitudinal measurement designs with multiple raters. The MR‐MLTS model can be estimated as a dynamic structural equation model using Mplus or the R‐package mlts, enabling researchers to incorporate external variables while accounting for individually varying time intervals between ratings.

The idiographic rater consistency coefficients are defined as variance ratio measures, akin to traditional ICCs used for assessing interrater reliability. However, the MR‐MLTS framework allows researchers to examine individual‐specific rater consistency coefficients and inter‐individual differences therein, free of measurement error. Rater consistency coefficients can be estimated for contemporaneous rater agreement, for time‐lagged consistency, consistency for the innovation components, as well as at the trait level. The contemporaneous rater consistency captures the overall contemporaneous convergence between two ratings within time points. The time‐lagged rater‐consistency coefficient captures the extent to which different rater groups (e.g., self‐ and partner ratings) exhibit consistency with respect to reliable, intra‐individual fluctuations that are carried forward in time. In contrast, the rater‐consistency for the innovation component reflects the degree of agreement with respect to unpredicted fluctuations over time, that is, in how far intra‐individual, unexplained fluctuations in the partner's ratings overlap with unexplained fluctuations in the self‐ratings. These coefficients offer a more granular understanding of how raters converge across varying temporal and situational contexts.

We illustrated the MR‐MLTS model using data from a German intensive longitudinal multirater study involving 100 heterosexual couples (or dyads) measured at 86 time points. In this study, the idiographic rater consistency coefficients can be interpreted as a direct measure of empathic accuracy, with higher rater consistency indicating a greater level of empathic accuracy. Our results reveal substantial individual differences in empathic accuracy, with coefficients ranging from .007 (no empathic accuracy) to .890 (high level of empathic accuracy). This variability underscores the importance of person‐specific approaches in understanding how individuals perceive and interpret their partners' momentary affective state over time.

Our results reveal substantial associations between rater consistency coefficients, suggesting that some couples consistently exhibit high (or low) levels of empathic accuracy across all positive affect items (i.e., happy, enthusiastic, and content). This finding indicates that empathic accuracy generalizes across different affect measures for certain individuals. To explore interindividual differences in empathic accuracy, we examined the relationship between rater consistency coefficients and external variables, specifically relationship duration, target's emotional resources, and partner's cognitive resources. For the innovations, higher rater consistency was significantly associated with relationship duration and the partner's cognitive resources. This suggests that partners who have spent some time in the relationship may be better at judging facets of momentary positive affect, specifically happiness and enthusiasm. Additionally, partners with greater cognitive resources demonstrated better accuracy in perceiving the target's momentary positive affect.

The results of our simulation study suggest that the reliability of individual parameter estimates is primarily driven by the number of observed time points per person, with very high reliability achieved at 200 observations per person. This indicates that, for accurate idiographic rater consistency estimates (reliability above .90), at least 200 time points are necessary. Fixed effect parameters are already accurately estimated in samples with 100 individuals and 100 time points. In contrast, accurate estimation of the between‐level random effect variances of the random model parameters requires a sample of at least 200 participants. Importantly, the results of our simulations should not be generalized beyond the conditions examined. Different sample size requirements can be expected under non‐normality, model misspecification, or increased model complexity, such as when adding additional rater groups, constructs, or random effects. It is strongly recommended that researchers undertake simulation studies to ascertain the minimum required sample size under various conditions. It is well known from the literature on Bayesian estimation of multilevel models that (a) random effect variances are prone to bias in small samples when using diffuse prior settings, and (b) small between‐level variances may hinder model convergence (e.g., Andriamiarana et al., 2023; Depaoli & Clifton, 2015; Schuurman, Grasman, & Hamaker, 2016). As small between‐level variances are prevalent in multilevel time series models, due to the bounds of random autoregressive effects in stationary models, the selection of adequate priors is essential. In general, incorporating prior information may help to improve the estimation accuracy. In our simulation study, we used the uninformative default priors as implemented in Mplus, which is a Inverse Wishart prior for the between‐level variance‐covariance matrix. Using mlts (Koslowski et al., 2024) or directly rstan or Stan (Stan Development Team, 2025a, 2025b) may improve estimation accuracy for the random effect variances and may be beneficial for fine‐tuning prior settings for variances and correlations of between‐level parameters, as rstan allows for the separation of the between‐level covariance matrix into its scales and a correlation matrix. Furthermore, using rstan offers the possibility for incorporating regularization methods (Andriamiarana et al., 2025; Jacobucci & Grimm, 2018). For other approaches, such as using admissible range restricted priors or data‐based prior specifications, see McNeish (2019) and Schuurman, Ferrer, et al. (2016).

Researchers may also compute idiographic rater consistency coefficients by fitting single‐level time‐series models separately for each dyad. However, our multilevel framework offers several advantages. By partially pooling information across dyads, it stabilizes estimates when individual time series are relatively short (Schultzberg & Muthén, 2018) and allows for a decomposition of within‐ and between‐dyad variance, thereby providing (a) insight into heterogeneity in rater consistency and (b) the possibility to compute rater consistency also at the trait level. In addition, estimating all dyads simultaneously within a single model ensures comparability of estimates and variance components, eliminating the need for post‐hoc aggregation. Nonetheless, single‐level time‐series models may be preferable when dyads have very long time series or when the primary focus is on case‐specific inference.

In future studies, researchers could investigate the extent to which hidden subgroups of dyads qualitatively differ in their idiographic rater consistency using cluster analysis, mixture modeling, or other unsupervised machine learning methods. Additionally, the model could be extended to multiple traits (e.g., positive and negative affect) or outcome variables such as mental or physical health. However, researchers should be aware that incorporating multiple traits into the MR‐MLTS model will substantially increase the number of free parameters.

Another avenue for future research is to extend the MR‐MLTS model to measurement designs that include random (interchangeable) raters, a combination of fixed and random raters, or other multirater structures such as round‐robin designs. These extensions could be implemented in a manner similar to the approaches discussed in the textbook by Eid et al. (2025) on multirater modeling.

## AUTHOR CONTRIBUTIONS

**Tobias Koch:** conceptualization; methodology; formal analysis; visualization; writing – original draft; writing – review and editing; software. **Miriam F. Jaehne:** writing – review and editing; methodology; conceptualization; writing – original draft. **Michaela Riediger:** writing – review and editing; data curation; funding acquisition. **Antje Rauers:** writing – review and editing; data curation. **Jana Holtmann:** conceptualization; writing – review and editing; software; formal analysis; visualization; methodology.

## DISCLOSURE OF ARTIFICIAL INTELLIGENCE‐GENERATED CONTENT (AIGC) TOOLS

The authors used ChatGPT, Deepl, as well as Overleaf writing assistant merely to improve spelling, grammar, and general editing. No AI assistant was used to write code. All code for the analyses was created by the authors. The authors reviewed the content generated and take full responsibility for the content of the article.

## CONFLICT OF INTEREST

All authors declared no conflict of interest. This research project was funded by the Deutsche Forschungsgemeinschaft (project number: 539550566).

## Supporting information


Table S1:

## Data Availability

The data that support the findings of this study are available at https://osf.io/g8hnz/?view_only=0df80fab09f74b249d4eeb7a56af1636, reference number osf.io/g8hnz. These data were derived from the following resources available in the public domain: https://osf.io/g8hnz/?view_only=0df80fab09f74b249d4eeb7a56af1636. The data that support the findings of this study are openly available on OSF at https://osf.io/g8hnz/?view_only=0df80fab09f74b249d4eeb7a56af1636, reference number osf.io/g8hnz.

## Footnotes

^1^
Note that with the current dataset, it is also possible to estimate an analogous model with the partners' self‐ratings of their positive affect as the target rating and the anchors' ratings of the partners' affect as the non‐reference (other) rating. The output of the respective model is also provided on OSF.

^2^
Plausible values are draws from the posterior distribution of the parameters. These draws are sampled as additional iterations after the requested burn‐in and post burn‐in iterations in Mplus. These values are thereby not identical to the post burn‐in iterations that are summarized in the Mplus Output. However, given that convergence was reached, the values are drawn from the same posterior distribution and should yield similar results. Note that in Mplus, the post‐burn‐in iterations of the random effects cannot be saved. Furthermore, we cannot directly sample the consistency coefficients within the model in Mplus, as it is up‐to‐date not possible to specify new parameters as functions of random effect parameters. We provide detailed R‐code to illustrate (a) the extraction of the plausible values, (b) the computation of the rater consistency coefficients per draw, and (c) their summary across draws. For reasons of consistency, we use a similar procedure for computing the coefficients when estimating the model with mlts and rstan, see example on OSF.

^3^
On OSF, we also provide code for the computation of the coefficients based solely on the person‐specific posterior medians of the parameters, as provided in the MPlus fscomparison output. Note, however, that we do not recommend this approach as it does not take into account the uncertainty in the parameter estimates and does not provide credibility intervals for the coefficients.

## References

[bmsp70022-bib-0001] Andriamiarana, V. , Kilian, P. , Brandt, H. , & Kelava, A. (2025). Are Bayesian regularization methods a must for multilevel dynamic latent variables models? Behavior Research Methods, 57(2), 71. 10.3758/s13428-024-02589-9 PMC11754388 39843620

[bmsp70022-bib-0002] Andriamiarana, V. , Kilian, P. , Kelava, A. , & Brandt, H. (2023). On the requirements of non‐linear dynamic latent class sem: A simulation study with varying numbers of subjects and time points. Structural Equation Modeling: A Multidisciplinary Journal, 30(5), 789–806. 10.1080/10705511.2023.2169698

[bmsp70022-bib-0003] Asparouhov, T. , Hamaker, E. L. , & Muthén, B. (2018). Dynamic structural equation models. Structural Equation Modeling: A Multidisciplinary Journal, 25(3), 359–388. 10.1080/10705511.2017.1406803

[bmsp70022-bib-0004] Blanke, E. S. , Rauers, A. , & Riediger, M. (2015). Nice to meet you—Adult age differences in empathic accuracy for strangers. Psychology and Aging, 30(1), 149–159. 10.1037/a0038459 25436599

[bmsp70022-bib-0005] Blanke, E. S. , Rauers, A. , & Riediger, M. (2016). Does being empathic pay off?—Associations between performance‐based measures of empathy and social adjustment in younger and older women. Emotion, 16(5), 671–683. 10.1037/emo0000166 26914335

[bmsp70022-bib-0006] Bohn, J. , Holtmann, J. , Ulitzsch, E. , Koch, T. , Luhmann, M. , & Eid, M. (2021). Analyzing stability and change in dyadic attachment: The multi‐rater latent state‐trait model with autoregressive effects. Frontiers in Psychology, 12, 604526. 10.3389/fpsyg.2021.604526 PMC8280502 34276461

[bmsp70022-bib-0007] Byrne, B. M. , Shavelson, R. J. , & Muthén, B. (1989). Testing for the equivalence of factor covariance and mean structures: The issue of partial measurement invariance. Psychological Bulletin, 105(3), 456.

[bmsp70022-bib-0008] Depaoli, S. , & Clifton, J. P. (2015). A bayesian approach to multilevel structural equation modeling with continuous and dichotomous outcomes. Structural Equation Modeling: A Multidisciplinary Journal, 22(3), 327–351. 10.1080/10705511.2014.937849

[bmsp70022-bib-0009] Eid, M. , Geiser, C. , & Koch, T. (2016). Measuring method effects: From traditional to design‐oriented approaches. Current Directions in Psychological Science, 25(4), 275–280. 10.1177/0963721416649624

[bmsp70022-bib-0010] Eid, M. , Geiser, C. , & Koch, T. (2025). Structural equation modeling of multiple rater data. Guilford Publications.

[bmsp70022-bib-0011] Eid, M. , Holtmann, J. , Santangelo, P. , & Ebner‐Priemer, U. (2017). On the definition of latent‐state‐trait models with autoregressive effects: Insights from LST‐R theory. European Journal of Psychological Assessment, 33(4), 285–295.

[bmsp70022-bib-0012] Eid, M. , Lischetzke, T. , Nussbeck, F. W. , & Trierweiler, L. I. (2003). Separating trait effects from trait‐specific method effects in multitrait‐multimethod models: A multiple‐indicator CT‐C (M‐1) model. Psychological Methods, 8(1), 38–60. 10.1037/1082-989X.8.1.38 12741672

[bmsp70022-bib-0013] Eid, M. , Nussbeck, F. W. , Geiser, C. , Cole, D. A. , Gollwitzer, M. , & Lischetzke, T. (2008). Structural equation modeling of multitrait‐multimethod data: different models for different types of methods. Psychological Methods, 13(3), 230–253. 10.1037/a0013219 18778153

[bmsp70022-bib-0014] Funder, D. C. (1999). Personality judgment: A realistic approach to person perception. Elsevier. 10.1037/0033-295x.102.4.652 7480467

[bmsp70022-bib-0015] Gwet, K. L. (2014). Handbook of inter‐rater reliability: The definitive guide to measuring the extent of agreement among raters. Advanced Analytics, LLC.

[bmsp70022-bib-0016] Hall, J. A. , Schmid Mast, M. , & West, T. V. (2016). The social psychology of perceiving others accurately. Cambridge University Press.

[bmsp70022-bib-0017] Hallgren, K. A. (2012). Computing inter‐rater reliability for observational data: an overview and tutorial. Tutorial in Quantitative Methods for Psychology, 8(1), 23–34. 10.20982/tqmp.08.1.p023 PMC3402032 22833776

[bmsp70022-bib-0018] Hamilton, J. (1994). Time series econometrics. Princeton University Press.

[bmsp70022-bib-0049] Holtmann, J. , & Koslowski, K. (2025). Modeling within‐level latent interaction effects in multilevel vector‐autoregressive models. Behavior Research Methods, 57, 277. 10.3758/s13428-025-02694-3 PMC12413427 40913220

[bmsp70022-bib-0019] Hu, Y. , Nesselroade, J. R. , Erbacher, M. K. , Boker, S. M. , Burt, S. A. , Keel, P. K. , Neale, M. C. , Sisk, C. L. , & Klump, K. (2016). Test reliability at the individual level. Structural Equation Modeling: A Multidisciplinary Journal, 23(4), 532–543. 10.1080/10705511.2016.1148605 PMC5603211 28936107

[bmsp70022-bib-0020] Ickes, W. (1997). Empathic accuracy. Guilford.

[bmsp70022-bib-0021] Jacobucci, R. , & Grimm, K. J. (2018). Comparison of frequentist and Bayesian regularization in structural equation modeling. Structural Equation Modeling: A Multidisciplinary Journal, 25(4), 639–649. 10.1080/10705511.2017.1410822 PMC6425970 30906179

[bmsp70022-bib-0022] Kenny, D. A. (2019). Interpersonal perception: The foundation of social relationships. Guilford Publications.

[bmsp70022-bib-0023] Koch, T. , Eid, M. , & Lochner, K. (2018). Multitrait‐multimethod‐analysis. In P. Irwing , T. Booth , & D. J. Hughes (Eds.), The Wiley handbook of psychometric testing (pp. 781–846). John Wiley & Sons, Ltd. 10.1002/9781118489772.ch25

[bmsp70022-bib-0024] Koch, T. , Voelkle, M. C. , & Driver, C. C. (2023). Analyzing longitudinal multirater data with individually varying time intervals. Structural Equation Modeling: A Multidisciplinary Journal, 30(1), 86–104. 10.1080/10705511.2022.2096612

[bmsp70022-bib-0025] Koslowski, K. , Münch, F. , Koch, T. , & Holtmann, J. (2024). Mlts: Multilevel latent time series models with R and Stan . https://github.com/munchfab/mlts 10.1037/met0000866 42804239

[bmsp70022-bib-0026] Lischetzke, T. , Eid, M. , Wittig, F. , & Trierweiler, L. (2001). Die wahrnehmung eigener und fremder gefühle. konstruktion und validierung von skalen zur erfassung der emotionalen selbst‐und fremdaufmerksamkeit sowie der klarheit über gefühle. Diagnostica, 47(4), 167–177. 10.1026/0012-1924.47.4.167

[bmsp70022-bib-0027] Malloy, T. E. (2018). Social relations modeling of behavior in dyads and groups. Academic Press.

[bmsp70022-bib-0028] McNeish, D. (2019). Two‐level dynamic structural equation models with small samples. Structural Equation Modeling: A Multidisciplinary Journal, 26(6), 948–966. 10.1080/10705511.2019.1578657 PMC7451754 32863699

[bmsp70022-bib-0029] McNeish, D. , Mackinnon, D. P. , Marsch, L. A. , & Poldrack, R. A. (2021). Measurement in intensive longitudinal data. Structural Equation Modeling: A Multidisciplinary Journal, 28(5), 807–822. 10.1080/10705511.2021.1915788 PMC8562472 34737528

[bmsp70022-bib-0030] Muthén, L. , & Muthén, B. (1998–2017). Mplus user's guide (8th ed.). Muthén & Muthén.

[bmsp70022-bib-0031] Putnick, D. L. , & Bornstein, M. H. (2016). Measurement invariance conventions and reporting: The state of the art and future directions for psychological research. Developmental Review, 41, 71–90. 10.1016/j.dr.2016.06.004 PMC5145197 27942093

[bmsp70022-bib-0032] R Core Team . (2024). R: A language and environment for statistical computing. R Foundation for Statistical Computing.

[bmsp70022-bib-0033] Rauers, A. , Blanke, E. , & Riediger, M. (2013). Everyday empathic accuracy in younger and older couples: Do you need to see your partner to know his or her feelings? Psychological Science, 24(11), 2210–2217. 10.1177/0956797613490747 24013188

[bmsp70022-bib-0034] Riediger, M. , & Blanke, E. S. (2020). Knowing what others think and feel: Empathic accuracy across adulthood. Annual Review of Developmental Psychology, 2(1), 157–176. 10.1146/annurev-devpsych-040920-035557

[bmsp70022-bib-0035] Savord, A. , McNeish, D. , Iida, M. , Quiroz, S. , & Ha, T. (2023). Fitting the longitudinal actor‐partner interdependence model as a dynamic structural equation model in mplus. Structural Equation Modeling, 30(2), 296–314. 10.1080/10705511.2022.2065279

[bmsp70022-bib-0036] Schultzberg, M. , & Muthén, B. (2018). Number of subjects and time points needed for multilevel time‐series analysis: A simulation study of dynamic structural equation modeling. Structural Equation Modeling: A Multidisciplinary Journal, 25(4), 495–515. 10.1080/10705511.2017.1392862

[bmsp70022-bib-0037] Schuurman, N. K. , Ferrer, E. , de Boer‐Sonnenschein, M. , & Hamaker, E. L. (2016). How to compare cross‐lagged associations in a multilevel autoregressive model. Psychological Methods, 21(2), 206–221. 10.1037/met0000062 27045851

[bmsp70022-bib-0038] Schuurman, N. K. , Grasman, R. , & Hamaker, E. (2016). A comparison of inverse‐wishart prior specifications for covariance matrices in multilevel autoregressive models. Multivariate Behavioral Research, 51(2–3), 185–206. 10.1080/00273171.2015.1065398 27028576

[bmsp70022-bib-0039] Schuurman, N. K. , & Hamaker, E. L. (2019). Measurement error and person‐specific reliability in multilevel autoregressive modeling. Psychological Methods, 24(1), 70–91. 10.1037/met0000188 30188157

[bmsp70022-bib-0040] Shrout, P. E. , & Fleiss, J. L. (1979). Intraclass correlations: Uses in assessing rater reliability. Psychological Bulletin, 86(2), 420–428. 10.1037/0033-2909.86.2.420 18839484

[bmsp70022-bib-0041] Stan Development Team . (2025a). RStan: the R interface to Stan [R package version 2.32.7] . https://mc‐stan.org/

[bmsp70022-bib-0042] Stan Development Team . (2025b). Stan Reference Manual, Version 2.37.0[Computer software] . https://mc‐stan.org/

[bmsp70022-bib-0043] Steyer, R. , Mayer, A. , Geiser, C. , & Cole, D. A. (2015). A theory of states and traits—revised. Annual Review of Clinical Psychology, 11(1), 71–98. 10.1146/annurev-clinpsy-032813-153719 25062476

[bmsp70022-bib-0044] ten Hove, D. , Jorgensen, T. D. , & van der Ark, L. A. (2022). Interrater reliability for multilevel data: A generalizability theory approach. Psychological Methods, 27(4), 650–666. 10.1037/met0000391 33818118

[bmsp70022-bib-0045] ten Hove, D. , Jorgensen, T. D. , & van der Ark, L. A. (2024a). Interrater reliability for interdependent social network data: A generalizability theory approach. Multivariate Behavioral Research, 60, 1–16. 10.1080/00273171.2024.2444940 39898488

[bmsp70022-bib-0046] ten Hove, D. , Jorgensen, T. D. , & van der Ark, L. A. (2024b). Updated guidelines on selecting an intraclass correlation coefficient for interrater reliability, with applications to incomplete observational designs. Psychological Methods, 29(5), 967–979. 10.1037/met0000516 36048052

[bmsp70022-bib-0047] Wechsler, D. (1994). Wechsler adult intelligence scale – revised (WAIS‐R). Psychological Corporation.

[bmsp70022-bib-0048] Xiao, Y. , Wang, P. , & Liu, H. (2023). Assessing intra‐ and inter‐individual reliabilities in intensive longitudinal studies: A two‐level random dynamic model‐based approach. Psychological Methods. 10.1037/met0000608 37561489

