# Supplementary material for: Idiographic interrater reliability measures for intensive longitudinal multirater data
Source: Br J Math Stat Psychol. 2025 Dec 20;79(3):547–68. doi: 10.1111/bmsp.70022 (PMC13679760; doi:10.1111/bmsp.70022)
Supplement: Supplementary file 1 [file BMSP-79-547-s001.pdf]

Supplemental material to:

Idiographic Interrater Reliability Measures for Intensive Longitudinal Multirater Data

Table S1: Between-level correlation matrix in the MR-MLTS model of the anchor's self-rated positive affect and the partner's other rating, including external between-level covariates.

	ϕ_O	$ln(\sigma^2_{\zeta_O})$	$\phi_O R$	$ln(\sigma^2_{\zeta_O R})$	λ happy	λ enth.	λ cont.	Rel.-dur.
ϕ_O	1	[-0.707;-0.302]	[-0.191;0.419]	[-0.315;0.245]	[0.113;0.639]	[0.083;0.612]	[0.044;0.578]	[-0.264;0.263]
$ln(\sigma^2_{\zeta_O})$	-0.536	1	[-0.481;0.071]	[0.195;0.633]	[-0.726;-0.242]	[-0.722;-0.247]	[-0.579;-0.025]	[-0.549;-0.115]
$\phi_O R$	0.12	-0.222	1	[-0.85;-0.541]	[-0.278;0.339]	[-0.437;0.155]	[-0.238;0.369]	[-0.132;0.401]
$ln(\sigma^2_{\zeta_O R})$	-0.039	0.438	-0.73	1	[-0.224;0.367]	[-0.214;0.356]	[-0.143;0.414]	[-0.568;-0.13]
λ happy	0.406	-0.526	0.035	0.08	1	[0.555;0.87]	[0.612;0.897]	[-0.195;0.343]
λ enth.	0.376	-0.521	-0.154	0.078	0.751	1	[0.294;0.745]	[-0.207;0.323]
λ content	0.334	-0.332	0.08	0.146	0.791	0.556	1	[-0.308;0.221]
rel.-dur.	0.001	-0.347	0.145	-0.369	0.079	0.06	-0.05	1
Emot. res. T	-0.066	0.151	0.082	0.111	-0.075	-0.12	0.032	-0.174
Cogn. res. P	-0.131	0.23	0.096	0.037	-0.02	-0.146	0.065	-0.602
μ enth. T	0.065	-0.231	0.069	-0.047	0.206	0.236	0.141	0.214
μ cont. T	-0.028	-0.066	0.005	0.058	0.141	0.051	0.038	0.315
μ happy P	0.078	-0.213	-0.113	0.038	0.113	0.18	0.117	-0.102
μ enth. P	0.112	-0.232	-0.009	-0.122	-0.017	0.228	0.029	0.121
μ cont. P	0.002	-0.26	-0.156	-0.023	0.222	0.284	0.044	0.162
μ happy T	0.003	-0.023	-0.013	-0.002	0.036	0.009	-0.028	0.192

Table continued

	Emot. res. T	Cogn. res. P	μ enth. T	μ cont. T	μ happy P	μ enth. P	μ cont. P	μ happy T
ϕ_O	[-0.323;0.205]	[-0.383;0.14]	[-0.212;0.334]	[-0.301;0.253]	[-0.208;0.346]	[-0.167;0.378]	[-0.271;0.273]	[-0.265;0.275]
$ln(\sigma^2_{\zeta_O})$	[-0.096;0.378]	[-0.015;0.451]	[-0.455;0.018]	[-0.32;0.192]	[-0.445;0.05]	[-0.457;0.026]	[-0.483;-0.008]	[-0.278;0.233]
$\phi_O R$	[-0.196;0.346]	[-0.176;0.355]	[-0.209;0.338]	[-0.272;0.286]	[-0.384;0.168]	[-0.285;0.268]	[-0.424;0.133]	[-0.285;0.262]
$ln(\sigma^2_{\zeta_O R})$	[-0.141;0.344]	[-0.212;0.277]	[-0.296;0.209]	[-0.206;0.31]	[-0.225;0.297]	[-0.362;0.139]	[-0.281;0.237]	[-0.254;0.252]
λ happy	[-0.347;0.197]	[-0.295;0.261]	[-0.086;0.458]	[-0.145;0.401]	[-0.182;0.388]	[-0.297;0.263]	[-0.06;0.473]	[-0.248;0.31]
λ enth.	[-0.379;0.155]	[-0.403;0.129]	[-0.049;0.48]	[-0.226;0.322]	[-0.106;0.443]	[-0.044;0.473]	[0.007;0.521]	[-0.267;0.284]
λ cont.	[-0.238;0.302]	[-0.214;0.33]	[-0.142;0.405]	[-0.234;0.304]	[-0.168;0.387]	[-0.248;0.3]	[-0.231;0.318]	[-0.296;0.247]
rel.-dur.	[-0.395;0.061]	[-0.735;-0.426]	[-0.026;0.432]	[0.076;0.517]	[-0.336;0.146]	[-0.123;0.349]	[-0.087;0.389]	[-0.058;0.414]
Emot. res. T	1	[-0.047;0.409]	[-0.064;0.404]	[-0.02;0.441]	[0.027;0.48]	[-0.019;0.439]	[-0.102;0.37]	[0.023;0.48]
Cogn. res. P	0.191	1	[-0.361;0.11]	[-0.384;0.096]	[-0.144;0.344]	[-0.383;0.089]	[-0.326;0.158]	[-0.328;0.156]
μ enth. T	0.178	-0.133	1	[0.354;0.703]	[0.063;0.516]	[0.037;0.489]	[-0.053;0.425]	[0.667;0.864]
μ cont. T	0.221	-0.154	0.552	1	[0.127;0.561]	[0.066;0.512]	[0.303;0.675]	[0.588;0.827]
μ happy P	0.267	0.105	0.308	0.363	1	[0.685;0.872]	[0.48;0.775]	[0.084;0.534]
μ enth. P	0.224	-0.156	0.278	0.307	0.796	1	[0.371;0.709]	[0.004;0.468]
μ cont. P	0.141	-0.089	0.195	0.513	0.649	0.562	1	[0.058;0.514]
μ happy T	0.267	-0.091	0.785	0.729	0.33	0.25	0.303	1

Note. Values below the diagonal denote (posterior medians of) between-level correlation coefficients, values above the diagonal their respective 95% credibility intervals.

Table S2: Relative parameter estimation bias, coverage rates, and mean squared error for the parameters of the MR-MLTS model in the simulation study, as estimated by MPlus.

	Relative parameter bias				95% Coverage Rate				MSE			
	$N = 100$		$N = 200$		$N = 100$		$N = 200$		$N = 100$		$N = 200$	
Time Points =	100	200	100	200	100	200	100	200	100	200	100	200
Parameter												
Fixed effects												
$\gamma_{\phi_{\mathrm{O}}}$	-0.052	-0.042	-0.049	-0.046	0.899	0.888	0.680	0.646	0.002	0.001	0.001	0.001
$\gamma_{\phi_{\mathrm{OR}}}$	-0.030	-0.022	-0.021	-0.021	0.950	0.969	0.940	0.970	0.001	0.001	0.000	0.000
$\gamma_{\lambda^w_{\mathrm{O12}}}$	-0.021	-0.019	-0.020	-0.026	0.980	0.990	0.960	0.909	0.002	0.001	0.001	0.001
$\gamma_{\lambda^w_{\mathrm{O22}}}$	-0.016	-0.016	-0.023	-0.021	0.970	0.990	0.970	0.960	0.002	0.001	0.001	0.001
$\gamma_{\lambda^w_{\mathrm{O32}}}$	-0.018	-0.014	-0.017	-0.019	0.990	0.990	0.960	0.950	0.002	0.001	0.001	0.001
$\gamma_{\ln(\sigma^2_{\zeta O})}$	-0.005	-0.015	-0.016	-0.018	1.000	0.959	0.960	0.960	0.003	0.003	0.002	0.002
$\gamma_{\ln(\sigma^2_{\zeta O})}$	-0.013	-0.004	0.000	-0.006	0.990	0.939	0.960	0.980	0.003	0.003	0.001	0.001
$\gamma_{\mu_{11}}$	-0.003	0.004	-0.002	0.004	0.970	0.969	0.920	0.939	0.013	0.015	0.006	0.007
$\gamma_{\mu_{12}}$	0.001	-0.001	0.002	0.009	0.970	0.959	0.980	0.990	0.012	0.011	0.005	0.004
$\gamma_{\mu_{21}}$	0.003	0.004	0.000	0.002	0.980	0.939	0.960	0.909	0.016	0.017	0.008	0.008
$\gamma_{\mu_{22}}$	-0.004	-0.003	-0.000	0.010	0.960	0.980	0.960	0.970	0.014	0.013	0.007	0.007
$\gamma_{\mu_{31}}$	0.001	0.004	-0.001	0.001	0.960	0.969	0.940	0.950	0.012	0.012	0.006	0.006
$\gamma_{\mu_{32}}$	-0.001	-0.000	0.001	0.004	0.970	0.990	0.950	0.980	0.011	0.010	0.005	0.004
Measurement model												
λ^w_O	0.008	0.003	0.004	0.001	0.924	0.929	0.965	0.960	0.000	0.000	0.000	0.000
λ^w_{OR}	-0.000	0.001	0.001	0.001	0.955	0.980	0.970	0.970	0.000	0.000	0.000	0.000
$\sigma^2_{\epsilon^w_{11}}$	0.007	0.003	0.002	0.001	0.950	0.949	0.930	0.970	0.000	0.000	0.000	0.000
$\sigma^2_{\epsilon^w_{21}}$	0.001	0.001	-0.001	0.001	0.990	0.949	0.950	0.990	0.000	0.000	0.000	0.000
$\sigma^2_{\epsilon^w_{31}}$	0.001	-0.000	0.000	0.001	0.980	0.959	0.960	0.970	0.000	0.000	0.000	0.000
$\sigma^2_{\epsilon^w_{12}}$	0.001	0.001	0.000	-0.001	0.929	0.939	0.890	0.929	0.000	0.000	0.000	0.000
$\sigma^2_{\epsilon^w_{22}}$	0.001	0.002	0.001	-0.001	0.980	0.969	0.960	0.960	0.000	0.000	0.000	0.000
$\sigma^2_{\epsilon^w_{32}}$	0.004	0.001	-0.002	-0.000	0.939	0.980	0.920	0.990	0.000	0.000	0.000	0.000
Random effect variances												
$\omega^2_{\phi_O}$	0.082	0.075	-0.089	-0.086	0.980	0.990	0.960	0.970	0.000	0.000	0.000	0.000
$\omega^2_{\phi_O R}$	0.333	0.307	0.083	0.080	0.697	0.745	0.940	0.950	0.001	0.001	0.000	0.000
$\omega^2_{\lambda^w_{\mathrm{O12}}}$	0.449	0.397	0.215	0.141	0.576	0.561	0.690	0.849	0.004	0.003	0.001	0.001
$\omega^2_{\lambda^w_{\mathrm{O22}}}$	0.493	0.442	0.218	0.144	0.515	0.561	0.740	0.899	0.004	0.004	0.001	0.001
$\omega^2_{\lambda^w_{\mathrm{O32}}}$	0.531	0.423	0.212	0.147	0.485	0.531	0.700	0.788	0.005	0.003	0.001	0.001
$\omega^2_{\ln(\sigma^2_{\zeta O})}$	0.445	0.347	0.159	0.112	0.646	0.643	0.850	0.869	0.010	0.006	0.002	0.001
$\omega^2_{\ln(\sigma^2_{\zeta OR})}$	0.427	0.410	0.195	0.159	0.556	0.571	0.780	0.838	0.010	0.008	0.002	0.002
$\omega^2_{\mu_{11}}$	0.468	0.447	0.221	0.217	0.434	0.490	0.570	0.646	0.275	0.285	0.079	0.068
$\omega^2_{\mu_{12}}$	0.420	0.433	0.212	0.229	0.485	0.531	0.660	0.576	0.280	0.394	0.064	0.069
$\omega^2_{\mu_{21}}$	0.443	0.448	0.199	0.199	0.455	0.469	0.640	0.657	0.373	0.383	0.093	0.083
$\omega^2_{\mu_{22}}$	0.438	0.431	0.201	0.213	0.444	0.408	0.630	0.596	0.460	0.471	0.082	0.087
$\omega^2_{\mu_{31}}$	0.495	0.468	0.217	0.224	0.394	0.408	0.610	0.606	0.255	0.256	0.062	0.057
$\omega^2_{\mu_{32}}$	0.426	0.470	0.211	0.222	0.495	0.398	0.640	0.566	0.246	0.399	0.052	0.052
